# Delivery Room Lung Ultrasound—Feasibility, Normal Patterns, and Predictive Value for Respiratory Support in Term and Near-Term Neonates: A Monocentric Study

**DOI:** 10.3390/life14060732

**Published:** 2024-06-06

**Authors:** Adrian Ioan Toma, Vlad Dima, Alina Fieraru, Alexandra Arghirescu, Larisa Nicoleta Andrășoaie, Răzvan Chirap, Anelise Alina Coandă, Teodora Bujdei, Andreea Nicoleta Marinescu, Al Jashi Isam

**Affiliations:** 1 Life Memorial Hospital, 010719 Bucharest, Romania; 2 Faculty of Medicine, University Titu Maiorescu, 040441 Bucharest, Romania; 3 Neonatology Department, Filantropia Clinical Hospital, 011132 Bucharest, Romania; 4 Neonatology Unit, Spitalul Clinic Municipal Filantropia, 200143 Craiova, Romania; 5 Faculty of Medicine, Carol Davila University of Medicine and Pharmacy, 050474 Bucharest, Romania

**Keywords:** lung ultrasound, delivery room, LUS score, LUS patterns, respiratory distress, CPAP

## Abstract

Aim: our study aimed to characterize the lung ultrasound (LUS) patterns noted immediately after delivery in term and near-term neonates, and to investigate whether the LUS scores or patterns observed at that point could anticipate the need for respiratory support in the sample of patients studied. Materials and methods: We performed two ultrasound examinations: one in the delivery room and the second at one hour of age. The anterior and lateral regions of both lungs were examined. We assessed the correlation between the LUS scores or patterns and the gestational age, umbilical arterial blood gases, the need for respiratory support (CPAP or mechanical ventilation), the presence of respiratory distress, and the need for the administration of oxygen. Results: LUS scores were significantly higher in the delivery room examination (8.05 ± 1.95) than at 1 h of age (6.4 ± 1.75) (*p* < 0.001). There were also statistically significant differences between the LUS patterns observed in different lung regions between the delivery room exam and the exam performed at 1 h of age (*p* values between 0.001 and 0.017). There were also differences noted regarding the LUS patterns between different lung regions at the exam in the delivery room (the right anterior region LUS patterns were significantly worse than the right lateral LUS patterns (*p* < 0.004), left anterior LUS patterns (*p* < 0.001), and left lateral LUS patterns (*p* < 0.001)). A statistically significant correlation was found between LUS scores and the gestational age of the patients (r = 0.568, *p* < 0.001—delivery room; r = 4.0443, *p* < 0.001—one hour of age). There were statistically significant associations between LUS scores, patterns at delivery (*p* < 0.001) and 1 h of age (*p* < 0.001), and the need for respiratory support (CPAP or mechanical ventilation). Conclusions: LUS in the delivery room offers important information regarding lung fluid elimination and aeration of the lungs, and early LUS features are significantly associated with the risk of respiratory distress and the need for respiratory support.

## 1. Introduction

Respiratory distress is the most frequent diagnosis for admission in the neonatal intensive care unit and the level of respiratory support needed is one of the most difficult decisions in neonatal intensive care [1,2,3]. The current guidelines recommend support to be administered as soon as possible (if possible, from the delivery room) and as less invasive as possible (CPAP or non-invasive ventilation) [4]. The early administration of surfactants, when indicated, is also considered essential for the management of the patient with respiratory distress [4].

Lung ultrasound is used more and more as an adjuvant in the neonatal intensive care unit in the care of neonates with respiratory pathology. It is very simple and easy to perform [5,6,7]. It is an ultrasound of artefacts, with the only real image being the pleural line and all the other lines being artefacts produced by the air–liquid interfaces [5,8]. It is used for the differential diagnoses of lung diseases in the newborn—transient tachypnea of the neonate [9], respiratory distress syndrome [10], neonatal pneumonia [11], meconium aspiration syndrome [12], pneumothorax [13,14], or pleural effusion [15]. Traditionally, a radiograph shows the air content of the lungs and the ultrasound shows the fluid content [8,10], so it seems tempting to use this method in the neonate, where there is a progressive post-natal aeration of the lungs and where the different diseases result from a lack of the development of this process. Also, scores have been established [16,17] to facilitate the decisions in the NICU and the appreciation of the evolution of the cases. There are scores established, with cutoff values suggesting the indication for surfactants, well correlated with the pressures used or with the oxygenation index [18]. A new multicenter study showed that a lung ultrasound score above 9 at one hour of age is a better and earlier indicator of the need for surfactant replacement therapy, especially when correlated with FiO_2_ [19].

Indeed, lung ultrasound performed at approximately one hour of life has been used with good results to identify the patients that will be admitted to the neonatal intensive care unit [16]. The white lung pattern, consisting of confluent A lines, has been correlated with a high probability of admission to the NICU and a high risk of the need for mechanical ventilation in term and near-term neonates [16]. The pattern consisting only of A lines with lung sliding was associated with a normal evolution [16]. Our group applied the same criteria in a similar validation study and the results were the same [18]. 

Could an earlier ultrasound evaluation establish earlier the need for admission to the NICU and the indication for respiratory support? This question is still open. 

The lungs become respiratory organs at birth, replacing the placenta as the organ for gas exchange [20,21]. To do this, the lungs must shift the air inside them—using airways and with the help of the respiratory muscles—and provide a good gas exchange surface. The establishment of lung aeration and functional capacity could be determined very well in the delivery room using lung ultrasound [22,23].

Studies using a modified scale from the one described in [16] have shown a progressive aeration of the lung during the first 20 min of life—with two phases—one establishing the lung volume—from an absent pleural line and a real image of fluid-filled lung (type 0) —to the established of the lung volume, heralded by the visualization of the pleural line (type 1) (with an intermediary type 0.5 present between 2–4 breaths—speckled pleural line before the establishment of the full pleural line) [22,23]. This process is finished in the normal neonate by the first 4 breaths. The second process is represented by lung aeration, with a progressive decrease of the fluid content as described also by Raimond. Type 1 has coalescent B lines (white lung appearance) showing the generalized presence of fluid [5,9] or exudate [5,10] with a limited gas exchange capacity. Type 2 has B lines present, but not con-fluent, and fluid is still present. Type 3 has A lines and lung sliding, with a normal appearance and fully aerated lungs [16,22,23]. According to this study, the whole process of lung aeration takes approximately 20 min to complete [22,23]. 

The authors of the above-mentioned study concluded that, based on their findings, lung ultrasound in the delivery room could be used to identify the neonates that need respiratory support or surfactant replacement therapy [22]. But is this the case? In a later study, the authors concluded that lung ultrasound (LUS) in the delivery room adequately predicts the need for surfactant therapy in premature infants with a gestational age of less than 32 weeks [24]. LUS performed at 5–10 min predicts better than FiO_2_ the need for surfactant replacement therapy in this category of neonates [24]. The problem is that in 50% of the cases, the phenomenon of backsliding is present; i.e., the lung ultrasound aspect worsens over time compared to the delivery room ultrasound [24]. This phenomenon could be due to several factors like progressive atelectasis due to fatigue of the neonate [24], the transition from active crying in the delivery room to a more regular pattern of respiration that does not distend the lungs so well [23] and has also been observed by others, and the establishment that backsliding occurs between 5–10 min and 2 h after delivery and no longer occurs after 2 h [23]. 

Based on the above-mentioned findings and considering our experience in the use of lung ultrasound spanning more than 10 years, we decided to perform a study on the use of lung ultrasound in the delivery room with the following primary objectives:

1. To characterize the lung ultrasound patterns noted immediately after delivery in term and near-term neonates.
2. To verify whether there is a correlation between the lung ultrasound patterns determined in the delivery room and the patterns observed at one hour of life.
3. To check if the lung ultrasound patterns observed immediately after delivery could anticipate the occurrence of respiratory distress, and the need for respiratory support in a sample of term and premature (≥33 weeks gestational age) neonates.

## 2. Materials and Methods

This was a prospective observational study performed in the delivery room and neonatal unit of Life Memorial Hospital, Bucharest. This study had the approval of the ethics committee of the hospital (Decision 1/20 February 2024), and the informed consent of the parents was obtained before the delivery.

Between 20 February and 10 April 2024, 100 patients were included in this study. We included consecutive deliveries until our sample reached 100 cases, so there were no specific inclusion criteria except the presence of the informed consent of the family before the delivery and gestational age > 33 weeks. The exclusion criteria were the absence of informed consent and delivery before 33 weeks of gestational age. In the period of study, 111 deliveries occurred at our hospital; in 5 cases, the families did not agree with the inclusion in this study; in 2 cases, the deliveries were emergencies and there was no time for an informed consent in these cases; and 4 neonates were born at gestational ages less than 33 weeks. 

### 2.1. Lung Ultrasound

Lung ultrasound examinations were performed with a portable device GE V-Scan Air (Vingmed Ultrasound AS, Horten, Norway), with a linear probe and frequency of 10 MHz. The ALARA principles were respected for lung ultrasound, with the MI (Mechanical Index) and TI (Thermal Index) less than 1 [25,26].

Two examinations were performed:

- The first examination was performed in the delivery room, after the delivery and stabilization of the neonate on the radiant warmer at 1–5 min after delivery.
- The second examination was performed with the same device at 1 h of life.

Both examinations consisted of images of the anterior and lateral regions of the lung as follows:

- Right lung anterior;
- Right lung lateral;
- Left lung anterior;
- Left lung lateral.

Films of 5–10 s were recorded with the probe placed in the sagittal plane. The ultrasound linear probe was placed first on the anterior chest, with the sign for right pointing upwards, followed by the axilla on the medial axillary line (right sign upwards) on the right side first and then on the left side. The same procedure was used for both delivery room and one-hour examinations. 

**Figure 1:**
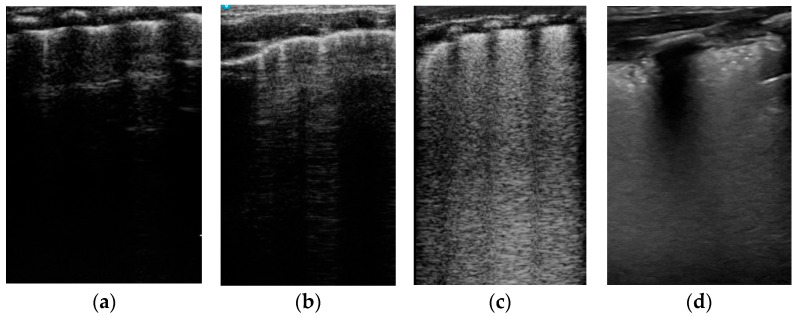
LUS score (Brat) [17]: (**a**) Score 0—only A lines and fewer than 3 B lines in the field. (**b**) Score 1—≥3 B lines in the field. (**c**) Score 2—white lung and coalescent B lines. (**d**) Score 3—extended sub-pleural consolidations.

Two types of lung ultrasound classifications were recorded:

**Lung ultrasound score (LUS score),** as described by Brat [17], assessed in 6 regions with a maximum score of 18 (Figure 1). We noted for each case the LUS score immediately after delivery and at 1 h of life. 

**Figure 2:**
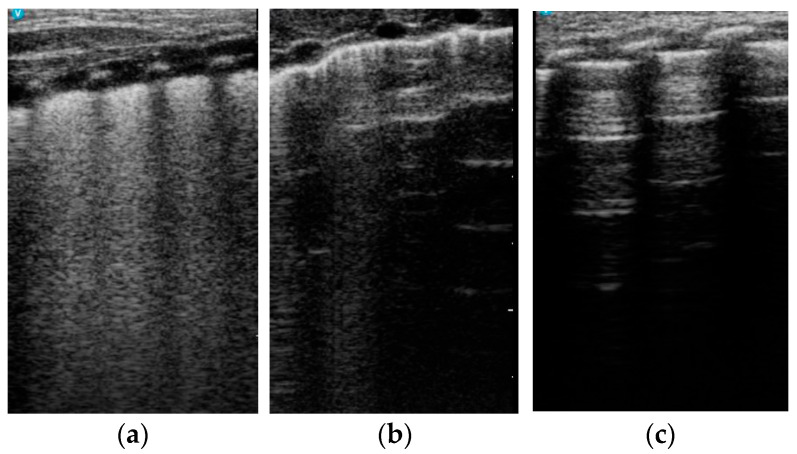
LUS patterns (modified from Raimondi [16]). (**a**) White lung. (**b**) Transition pattern with A line and B lines present. (**c**) Normal pattern with A lines, <3 B lines, and lung sliding.

The images were also classified according to **a modified version of the system proposed by Raimondi [16]** into three categories:

- White lung and confluent B lines (type 1 Raimondi) (Figure 2a);
- Transition pattern—A and B lines visible—B lines do not confluate (type 2 Raimondi) (Figure 2b)
- Normal lung with A lines, rare B lines, and lung sliding (type 3 Raimondi) (Figure 2c).

The decision to assign denominations instead of a number was taken because these are discontinuous variables and we wanted to compare the incidence of each pattern in different regions and moments in time, and a numeric designation would have created confusion with the scores. 

The patterns for all the 4 regions were noted for every patient. The person performing the ultrasound was not part of the resuscitation team and did not participate in the delivery of care to the neonate. The ultrasound did not interfere with the care of the neonate; the results were not available to the caretaker and did not influence the process of decision making. However, the LUS was performed independently at 1 h of age with another ultrasound device as part of the decision-making process for the neonate with respiratory distress, according to the internal protocol.

### 2.2. Other Variables

To establish correlations, other variables were collected from the patients’ records before discharge:

- Gestational age and birth weight.
- The delivery room blood gas values.
- The need for positive-pressure ventilation in the delivery room.
- The presence of respiratory distress.
- The need for mechanical ventilation or CPAP.

The decision for resuscitation, admission to the NICU, and respiratory support of the patient was the clinician’s responsibility and was in accordance with the internal protocol, developed after the AAP and ERC neonatal resuscitation guidelines [27,28] and the RDS treatment consensus guidelines [4]. 

We also collected data related to the history of pregnancy and the course of the baby in the nursery:

- Presence of maternal diabetes or pregnancy-induced hypertension;
- Smoking in the mother;
- Mode of delivery—cesarean section versus spontaneous vaginal delivery;
- Risk of materno-fetal infection (positive vaginal cultures of the mother or urinary tract infection in the mother during pregnancy);
- Color of the amniotic fluid at delivery;
- Use of antibiotics in the neonate;
- Need of IV line in the neonate (glucose or parenteral nutrition);
- Duration of stay in hospital of the neonate.

### 2.3. Statistical Analysis

We compared LUS scores at delivery and at 1 h, lung ultrasound patterns in different lung regions (right anterior, right lateral, left anterior, left lateral) immediately after delivery and at 1 h, and significant differences between the lung ultrasound patterns in different lung regions immediately after delivery (right anterior versus right lateral versus left anterior versus left lateral) and at 1 h.

We also assessed the correlation between the values of LUS scores (at delivery and 1 h), lung ultrasound patterns (at delivery and 1 h), the gestational age of the baby, the values of the blood gases at delivery, the data related to the history of the pregnancy (smoking in the mother, maternal diabetes or pregnancy-induced hypertension, risk of materno-fetal infection), the mode of delivery (stratified for gestational age), the color of the amniotic fluid at delivery, and certain features of the clinical course of the neonate (need of an IV line, use of antibiotics, duration of stay in the hospital).

Enhanced attention was dedicated to the analysis of the relation between lung ultrasound findings and the need for respiratory assistance or support of the neonate. We assessed the correlation between LUS scores immediately after delivery and at 1 h, the lung ultrasound patterns immediately after delivery and at 1 h, and the need for the use of oxygen, CPAP, or mechanical ventilation.

The statistical methods used were chosen depending on the types of variables. To assess the statistically significant difference between two or more groups, depending on the distribution of the series of values, for a significance of 95% for the numeric continuous variables, we applied the Student’s *t*-test—a parametric test that compares the mean values in two groups with a normal distribution of the values. The Chi-Square Test, a non-parametric test, was used to compare one or more frequency repartitions from the same population when the expected events are excluding one another—for a small frequency, the Yates Correction was applied. We used the Kruskall–Wallis test for rank differentiation of one or more independent samples—this could be considered an analysis of variance for ordinal data. An association was considered statistically significant if the *p*-value was smaller than 0.05. The “Pearson” correlation coefficient was used to assess the direct or indirect correlation between two variables from the same sample [29,30].

## 3. Results

Between 20 February and 10 April 2024, 100 neonates were included in this study. The mean gestational age of the patients was 38.25 weeks (+1.35 weeks) with a minimum of 33 and a maximum of 41 weeks. There were 58 male neonates and 42 female neonates. 

The incidence of different conditions in the mother and infant can be analyzed in Table 1.

### 3.1. Characterization of Lung Ultrasound Scores and Patterns in the Delivery Room and at 1 h of Age

#### 3.1.1. LUS Score

The LUS score values in the delivery room and at 1 h of age have a homogenous distribution; consequently, non-parametric tests for significance were applied. For rank differentiation, the Wilcoxon test was applied. 

LUS score values in the delivery room were significantly higher than the LUS values at 1 h of age (*p* < 0.001) (Table 2)

#### 3.1.2. LUS Patterns

**Figure 3:**
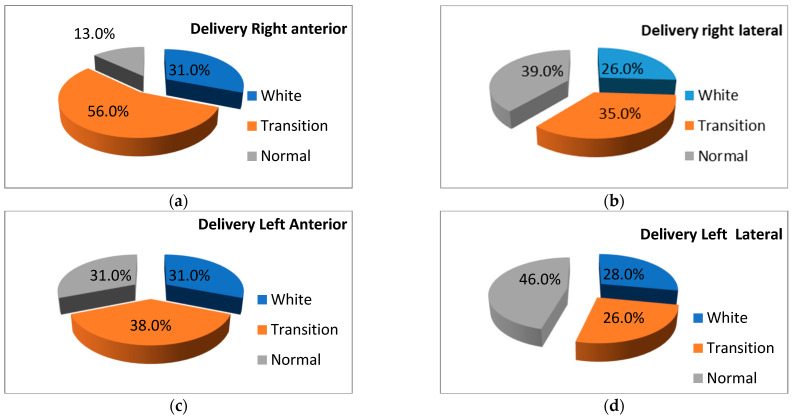
Delivery room LUS patterns: different lung regions for the definition of patterns (see Materials and Methods and Figure 2). Legend (LUS—lung ultrasound). Each figure has as a title the region of the lung that was assessed (see text) and as color codes the LUS patterns observed.

**Figure 4:**
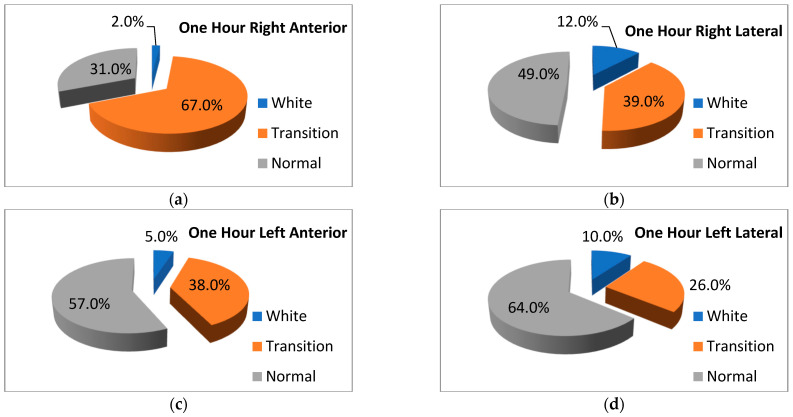
LUS patterns at 1 h: different lung regions. Legend—LUS: lung ultrasound Each figure has as a title the region of the lung that was assessed (see text) and as color codes the LUS patterns observed.

The frequencies for different LUS patterns can be followed in Figure 3 (delivery room LUS) and Figure 4 (LUS at 1 h). 

At the delivery room LUS, right anterior LUS patterns were significantly worse than right lateral LUS patterns (*p* < 0.004), left anterior LUS patterns (*p* < 0.001), and left lateral LUS patterns (*p* < 0.001). The right lateral pattern was significantly different from the left anterior (*p* < 0.002) and left lateral (*p* < 0.001) patterns. The left anterior LUS patterns were significantly worse than the left lateral LUS patterns (*p* < 0.001). 

Statistically significant differences were noted between the LUS patterns observed in different regions of the lungs at 1 h: right anterior vs. right lateral (*p* < 0.001), right anterior vs. left anterior (*p* < 0.001), right anterior vs. left lateral (*p* < 0.013), right anterior vs. left anterior (*p* < 0.001), right lateral vs. left lateral (*p* < 0.001), and left lateral vs. left anterior (*p* < 0.017). 

In all these cases, the Pearson Chi-Square Test was used. 

### 3.2. Association of the LUS Scores and Patterns with Different Conditions

**Figure 5:**
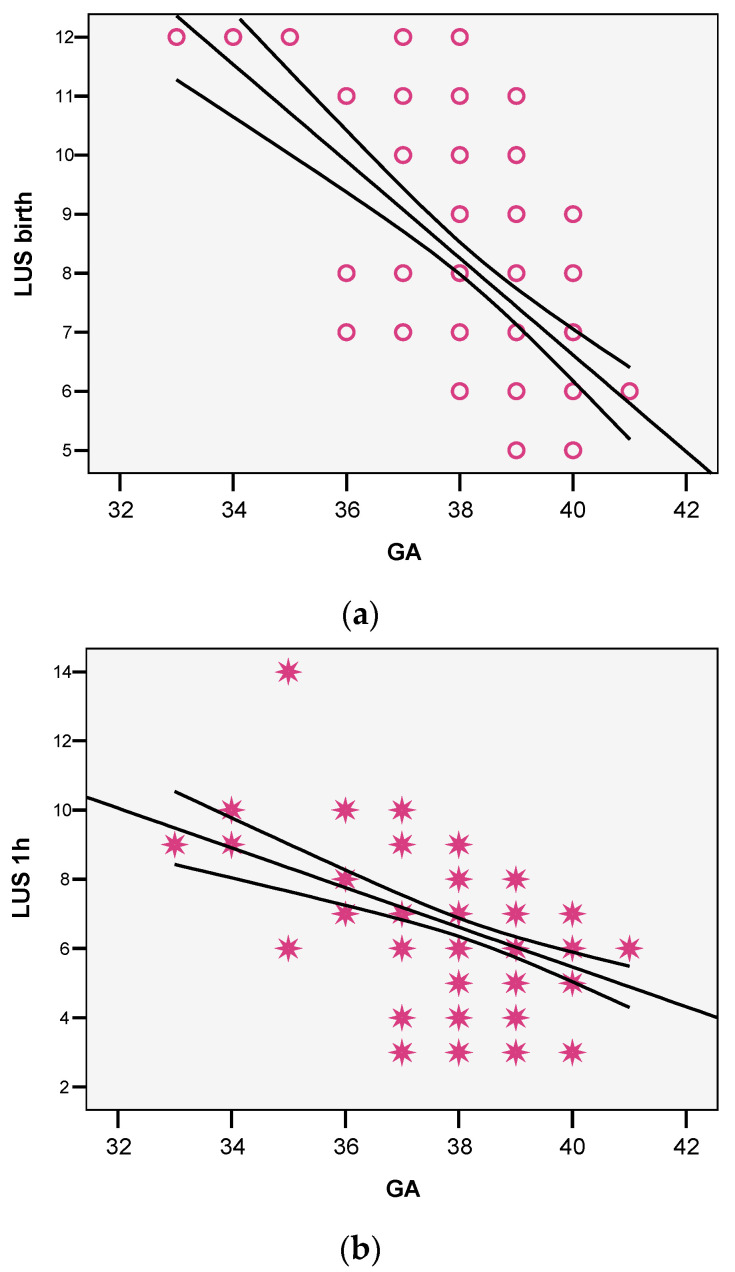
(**a**) Correlation between LUS score at delivery and gestational age. (**b**) Correlation between LUS score at 1 h and gestational age. Legend (GA = gestational age; LUS—lung ultrasound (score)). The bullets in (**a**) and the stars in (**b**) represent individual patients.

A strong association was found between the gestational age of the infant and the LUS score in the delivery room and at 1 h (Figure 5a,b) (r = 0.568, *p* < 0.001—delivery room; r = 4.0443, *p* < 0.001—one hour of age). 

There was no statistically significant correlation between LUS scores at delivery or at one hour and the values of arterial umbilical blood gases collected at delivery (Table 3).

LUS scores at delivery did not differ significantly between neonates born by cesarean section versus vaginal delivery (*p* < 0.119); this pattern persisted also at one hour (*p* < 0.510).

Among the conditions affecting the mother and the neonate, in the case of LUS score in the delivery room and at one hour, the only significant association was found in the case of antibiotic treatment in the neonate (*p* < 0.029) (Table 4).

Concerning the duration of stay in the hospital, there is a direct correlation with moderate statistical significance between an increased LUS score at delivery (r = 0.451 *p* < 0.001) and at 1 h (r = 0.373 *p* < 0.001) and an increased duration of hospitalization. 

### 3.3. Correlation between LUS Scores and LUS Patterns and Respiratory Conditions: Prediction of Respiratory Support

There was a strong correlation between LUS scores in the delivery room and at 1 h and the risk of CPAP (Table 5)

Also, there was a statistically significant association between the LUS patterns in the delivery room and at 1 h and the risk of CPAP (Table 6)

There was also a statistically significant association between the risk of administration of oxygen (Table 7) and respiratory distress (Table 8) and the LUS scores in the delivery room and one hour. 

Since only three patients were mechanically ventilated among those that were initially on CPAP, no statistical analysis was possible for this group. All these patients had an LUS score of 12 in the delivery room. In one of the cases, backsliding was noted, with an LUS score of 14 at one hour; in two of the cases, the score remained constant (score of 12) despite CPAP use in the delivery room. 

## 4. Discussion

Our research fulfilled its aim of characterizing the lung ultrasound patterns for both anterior and lateral lung fields in the delivery room and comparing these patterns with the findings in similar regions 1 h after delivery. 

Previous studies [22,23,24] described the features of LUS just in the lateral lung fields in the delivery room, on the axillary line. We chose to follow another direction, like a study published by Bhatia and co-workers in 2021, that assessed both the anterior and lateral lung fields [31], because we believed that much information could be gained from studying the anterior lung fields.

We chose the moment for assessment for practical reasons and regarding the timing for the filling of the lungs with air. Previous studies [22,23] investigated the neonatal lung fields immediately after delivery, and much information was obtained—including the timing of the establishment of the pleural line, the time to full lung aeration, etc. [22,23]. The procedure is difficult and, even if it is appropriate in a research setting, could be impractical in the everyday life in the hospital and delivery room. In addition, studies found a good correlation between the pattern at 1–5 and 5–10 min and the need for ventilation and the administration of surfactants [22,23,24]. So, we decided to perform the ultrasound examination in the first 5 min, with a stable infant, when the person responsible for the care of the baby decided to allow this. In this way, we could have a homogenous sample, we obtained valuable information early enough, and the procedure would be better adopted in the general practice. Performing the ultrasound examination during the first 30 min after delivery [31], even if this could mean having more stable lungs from the point of view of the aeration, could delay the establishment of, for example, a CPAP in the delivery room or early surfactant administration. Also, our study showed that the early examination is better correlated with the risk of oxygen administration and respiratory support than the exam at 1 h, even if they both were statistically significant. 

Another practical lesson that we learned in this study is that an extra person should perform the LUS evaluation, to avoid disturbing the clinician in the care of the infant. This could complicate a little bit the applicability of the protocol, but it is our belief that this would bring more safety and stability to the patient. 

We used two scoring systems to assess the LUS findings—the Brat score and a modified version of the Raimondi classification of lung patterns. We think that this could be a strong point of our study because we provided delivery room data for the two systems and compared them with the LUS appearance at one hour. We decided to use denominations instead of numbers for the Raimondi classification to strengthen the fact that these are not continuous but qualitative variables, and the three patterns should be compared between different regions. 

We first discuss the findings regarding the LUS score. We established that there was a statistically significant difference in the score recorded in the delivery room and that recorded at 1 h, with the scores in the delivery room being significantly higher. This finding was to be expected if we consider that the lungs begin to progressively fill with air [20,32,33] after delivery and that the lung ultrasound is based on air–fluid artefacts [22,23,34,35], so progressive filling with air will decrease the number of B lines and their density, progressively lowering the score.

There was no statistically significant association between the LUS scores at delivery and at 1 h and the arterial cord blood gas values. This could also be logical because arterial blood gas values reflect the fetal oxygenation [36] and the lungs are not involved in the process of oxygenation in fetal life [37].

The strong association between the LUS score in the delivery room and the gestational age found in our study is probably related to the immaturity of the lung present even at gestational ages over 33 weeks and before term and to the delayed clearance and persistent secretion of fetal lung fluid even at later gestational ages before term [38,39]. This association is present also in the case of the LUS score at one hour.

Among the other variables studied, not including those related to respiratory status, a statistically significant association was found in the case of the treatment with antibiotics in the nursery and the length of stay in the hospital. We believe that this association is determined by confounders (i.e., respiratory distress or respiratory support) because the neonates with respiratory distress or those being ventilated are more prone to treatment with antibiotics and prolonged hospitalization. It is interesting that we did not find a statistically significant association between the lung score and the mode of delivery. Several studies found this association to be present [22,23], while others did not [31]. We think that the small number of vaginal deliveries in this study could represent a bias in finding this kind of association.

Even if the scoring system proposed by Raimondi seems simpler than the LUS score, it provided us with valuable data regarding the filling of the lungs with air after delivery, because it allowed us to characterize separately each lung region and to assess the differences between regions during the same examination and the evolution of the pattern in each region at different moments in time.

We found statistically significant differences between the LUS patterns in the different regions of the lung at the exam performed in the delivery room (Figure 3), with the anterior regions of both lungs exhibiting the worst pattern (31% white lung). There could be two explanations for this. The first one resides in the timing of the examination of the lung regions. We began with the right anterior region, followed with the right lateral, left anterior, and left lateral regions. Each exam consisted of a 5–10 s film. Previous studies [22,23] described modifications of the lung pattern from one breath to the next during the first minutes of life, so the fact that we did not examine both regions of the lung at the same time could be the reason for this difference—with the regions examined later exhibiting a better pattern. There is an observation to this: the anterior right and left regions have the same percentage of white lung patterns, different from the lateral regions of the same lung. The right lateral region has a better pattern than the left anterior region, even if it is examined earlier. So, we truly believe that the explanation for this is related to the progressive aeration of the lungs. Animal studies describe the progressive aeration of the lung, with progressive and subsequent inspiratory movements pushing the liquid into the interstitial space; the aeration begins in the large proximal airways and ends in the distal airways and alveoli, near the diaphragm [32]. Also, after delivery, the diaphragm contracts and deforms the chest walls at the points of insertion, limiting the expansion of those regions for the first couple of minutes [40]. After birth, the stiffening of the chest walls would decrease the deformation induced by the contraction of the diaphragm, allowing these areas to aerate [41,42]. Another mechanism could also play a role—the expansion of the air occurs during the inspiratory phase; during the expiratory phase, the fluid accumulates from the airways and the oral cavity and pharynx, especially during the first minutes [32]. Also, it has been shown that the apical regions aerate less and more slowly during spontaneous breathing [32,33]. All these mechanisms could determine the anterior regions of the lung to be less aerated than the lateral regions during the first minutes of life, in the spontaneously breathing neonate, as observed in our study.

Regarding the need for respiratory support, both the LUS scores and the LUS patterns in the delivery room were significantly associated with the incidence of respiratory distress, the use of oxygen, and the need for CPAP. There was also a statistically significant association between the same variables and the LUS scores and LUS patterns at 1 h. Indeed, the neonates who needed respiratory support (CPAP or mechanical ventilation) had significantly higher LUS scores than the infants who did not need CPAP. In the case of the LUS patterns, the most significant association was again that of a worse LUS pattern in the anterior lung fields; an anterior white lung pattern (either on the left or right side) was a strong predictor of the need for CPAP. The prediction of ventilator support and of surfactant administration was also found in other studies [24,31], and our study has the particularity of assessing both the lung score and the lung LUS patterns and refining the findings by indicating which one of the lung regions has a better predictive value. Based on these data, we consider that LUS could be used as an aid to establish the need for respiratory support in the delivery room. Indeed, even if there have been discussions about the superiority of a clinical score in the delivery room to establish the need for positive pressure and surfactant administration [43]—considered better than LUS because LUS needs a new device to be operated in the delivery room, needs a trained person, and needs more time spent in evaluation—LUS is a more objective parameter, is consistent, comes with a good predictive value as demonstrated by other studies both in the delivery room [24,31] and during the first hours of life [6,33,34,44], could result in earlier respiratory support and surfactant administration [44], and could improve the prognosis of the neonates. Maintaining a good respiratory status in premature neonates could also result in improved general homeostasis and the avoidance of important neurologic complications [45].

The small number of infants in need of mechanical ventilation (3 patients) in this cohort represents a weak point of this study, not allowing the establishment of a correlation between LUS, the need for mechanical ventilation, and the need for surfactant administration.

In our opinion, the strong points of this study are its prospective design, the sufficiently large sample size, the unity of practice (same delivery room, same unit, same staff), the use of both the LUS score and LUS patterns, and the characterization and comparison of separate lung regions in the delivery room and at one hour. The design of the approach, allowing stabilization of the infant before performing the ultrasound, but not delaying it too much, makes the protocol more applicable in the clinical setting, which could be considered another strong point of this study. Another strong point of this study, related to the study design, is that the clinical decisions for respiratory support were not related to the results of LUS (the clinician was not aware of our delivery room and 1 h assessment, and the first ultrasound was performed by the healthcare provider at 1 h of age, with another device).

We also identified several weak points and limitations in our research that could diminish its value and applicability. The first weak point is the lack of small premature infants and of a greater number of infants with severe conditions needing mechanical ventilation. Even if the unity of practice and the single-center study could represent a strong point, this could also represent a weak point in the way that a multi-centric study could make the results more valuable and applicable in general practice. Also, a multi-centric study would prove that the protocol and the results are reproducible in different settings. Another weak point related to the study population is represented, as previously stated, by the small number of vaginal deliveries that limit our capability to assess whether the vaginal delivery results in better aeration of the lung, thus limiting the value of our findings. Also, after analyzing the data, we believe that we should have included data about the aspiration at delivery, to see whether the lack of aspiration or the presence of excess fluid is related to worse lung aeration during the first minutes. Another weak point could be represented by the lack of intermediate LUS evaluations between the delivery room and 1 h, and of further LUS evaluations during the first 24 h to evaluate the progression of lung aeration. We tried though to keep the design as simple as possible and to not duplicate another study conducted in this field [12,22,23,24].

Based on all of the above, we consider that our next directions of research could be the following:

- To investigate whether the LUS pattern differences noted at delivery are not due to different moments of examination by concomitant examination with two devices of different lungs and lung regions in the same patient.
- To take into account in a future delivery room LUS study other variables that could influence the clearance of lung fluid in different regions—position of the baby, presence of secretions in the airways, and aspiration of the secretions.
- To assess whether there are statistically significant differences between neonates born vaginally and by cesarean section by recruiting a larger cohort.
- To assess whether the LUS scores and LUS patterns at delivery are also good predictors for mechanical ventilation and indications for surfactant replacement therapy.
- To investigate whether the use of LUS as an adjuvant for decision making in these situations could result in a better prognosis for infants with respiratory distress.

We believe that neonatal ultrasound, due to its ease of use and non-invasiveness, can even become a screening method. This, however, must be implemented on the basis of larger studies, possibly multicenter, and which also includes groups of premature patients, a fact already mentioned in the paragraph discussing the weak points and limitations of this study.

## 5. Conclusions

Our study showed that the LUS score is significantly higher at the examination performed in the delivery room than at 1 h of life in term and near-term newborns, and there is a statistically significant difference between the LUS patterns present at delivery and the patterns at one hour of life in the same category of infants, probably showing the progressive aeration of the lungs. LUS patterns are worst in the anterior regions of both lungs at delivery, giving us insight into the mechanism of lung fluid elimination and lung aeration during the first minutes of life. Both higher LUS scores and worse LUS patterns (white lungs) at delivery and one hour were significantly associated with the risk of respiratory distress, risk of oxygen administration, and use of CPAP. Thus, we conclude that LUS in the delivery room is feasible and offers important information regarding lung fluid elimination and aeration of the lungs, and early LUS features could lead to an earlier decision regarding the respiratory support of the neonates, resulting in an improvement in their medical care.

Further studies are needed to confirm these findings in a larger population and to assess whether the treatment performed based also on LUS features could result in a better prognosis and fewer complications for the patients

## Figures and Tables

**Table 1:** Incidence of different conditions in the group.

Condition	Number	Percentage
Gestational diabetes (mother)	11	11%
Pregnancy-induced hypertension (mother)	4	4%
Smoking (mother)	19	19%
Risk of materno-fetal infection	38	38%
Meconial amniotic fluid	7	7%
Delivery by cesarean section/vaginal	93/7	93%/7%
IV lines in the neonate	24	24%
Antibiotic treatment in the neonate	19	19%
Respiratory distress	13	13%
Administration of oxygen	10	10%
CPAP	9	9%
Mechanical ventilation	3	3%

Legend: CPAP—continuous positive airway pressure; IV lines—intravenous line.

**Table 2:** LUS values in the delivery room and at 1 h of age (LUS—lung ultrasound).

	Mean	Standard Deviation	Median	Variance	Minimum/Maximum
Delivery room	8.05	1.95	8	3.81	5/12
1 h	6.4	1.75	7	3.06	3/14

**Table 3:** Correlation between LUS scores and umbilical arterial blood gases at delivery.

		LUSbirth	LUS 1h	pH	pCO_2_	pO_2_	BE	HCO_3_^−^
LUS score	Pearson Correlation	1	0.473 **	−0.082	0.059	−0.104	0.018	−0.010
Birth	Sig. (2-tailed)		0.000	0.418	0.557	0.306	0.862	0.922
	N	100	100	100	100	99	100	100
LUS score	Pearson Correlation	0.473 **	1	0.008	−0.111	−0.021	−0.130	−0.119
1h	Sig. (2-tailed)	0.000		0.940	0.272	0.838	0.196	0.239
	N	100	100	100	100	99	100	100
pH	Pearson Correlation	−0.082	0.008	1	−0.598 **	0.339 **	0.353 **	0.621 **
	Sig. (2-tailed)	0.418	0.940		0.000	0.001	0.000	0.000
	N	100	100	100	100	99	100	100
pCO_2_	Pearson Correlation	0.059	−0.111	−0.598 **	1	−0.388 **	0.397 **	0.020
	Sig. (2-tailed)	0.557	0.272	0.000		0.000	0.000	0.844
	N	100	100	100	100	99	100	100
pO_2_	Pearson Correlation	−0.104	−0.021	0.339 **	−0.388 **	1	−0.013	0.251 *
	Sig. (2-tailed)	0.306	0.838	0.001	0.000		0.900	0.012
	N	99	99	99	99	99	99	99
BE	Pearson Correlation	0.018	−0.130	0.353	0.397 **	−0.013	1	0.909 **
	Sig. (2-tailed)	0.862	0.196	0.000	0.000	0.900		0.000
	N	100	100	100	100	99	100	100
HCO_3_^−^	Pearson Correlation	−0.010	−0.119	0.621 **	0.020	0.251 *	0.909 **	1
	Sig. (2-tailed)	0.922	0.239	0.000	0.844	0.012	0.000	
	N	100	100	100	100	99	100	100

* Correlation is significant at the 0.05 level (2-tailed); ** Correlation is significant at the 0.01 level (2-tailed); Legend: LUS—lung ultrasound; 1 h—one hour; pCO_2_—arterial pressure of carbon dioxide; pO_2_—arterial pressure of oxygen; BE—base excess; HCO_3_^−^—bicarbonate.

**Table 4:** Correlation between LUS score values and different maternal and neonatal conditions.

Parameters	LUS Score Birth		LUS Score 1 h	
	Mean Rank	p	Mean Rank	*p*
Maternal diabetes	63.32 48.92	0.115	51.27 50.40	0.924
Pregnancy-induced hypertension	69.00 49.73	0.204	37.63 51.04	0.379
Risk of maternofetal infection	48.74 51.58	0.629	54.78 47.88	0.238
Amniotic fluid—meconial	44.93 50.92	0.598	48.79 50.63	0.868
Male Female	50.18 50.94	0.896	48.09 53.83	0.317
**Antibiotics yes/no**	**66.58** **46.73**	**0.006**	**63.26** **47.51**	**0.029**

Legend: LUS—lung ultrasound.

**Table 5:** LUS score and the prediction of CPAP.

LUS Score	CPAP			Non-CPAP			* *p*
	Mean ± SD	Median	Limits	Mean ± SD	Median	Limits	
Delivery room	11.44 ± 1.33	11	8–12	7.71 ± 1.66	8	5–11	0.001
One hour	8.33 ± 3.00	8	3–14	6.29 ± 1.48	6	3–10	0.001
* *p*	0.004			0.001			

* *p* values for *t*-Student test. Legend: LUS—lung ultrasound; CPAP—continuous positive airway pressure; SD—standard deviation.

**Table 6:** Association between LUS patterns and risk of CPAP (LUS—lung ultrasound; CPAP—continuous positive airway pressure).

	Pattern				
	Region	White	Transition	Normal	*p* Value
Delivery room	Right anterior	9 (100%)	-	-	0.001
	Right lateral	7 (77.8%)	1 (11.1%)	1 (11.1%)	0.003
	Left anterior	8 (88.9%)	1 (11.1%)	-	0.001
	Left lateral	7 (77.8%)	1 (11.1%)	1 (11.1%)	0.004
One hour	Right anterior	1 (11.1%)	8 (88.9%)	-	0.013
	Right lateral	4 (44.4%)	5 (55.6%)	-	0.001
	Left anterior	1 (11.1%)	6 (66.7%)	2 (22.2%)	0.081
	Left lateral	4 (44.4%)	3 (33.3%)	2 (22.2%)	0.005

**Table 7:** Association between LUS scores and need for oxygen.

LUS Score	Oxygen Needs			No Oxygen			* *p*
	Mean ± SD	Median	Limits	Mean ± SD	Median	Limits	
Delivery room	10.50 ± 1.43	10	8–12	7.78 ± 1.81	8	5–12	0.001
One hour	7.70 ± 2.83	8	3–14	6.33 ± 1.55	6	3–10	0.018
* *p*	0.002			0.001			

* *p* values for *t*-Student test. Legend: LUS—lung ultrasound; SD—standard deviation.

**Table 8:** Association between LUS scores and risk of respiratory distress.

LUS Score	Respiratory Distress			No Respiratory Distress			* *p*
	Mean ± SD	Median	Limits	Mean ± SD	Median	Limits	
Delivery room	11.00 ± 1.41	11	8–12	7.61 ± 1.61	8	5–12	0.001
One hour	7.85 ± 2.73	8	3–14	6.269 ± 1.47	6	3–10	0.002
* *p*	0.001			0.001			

* *p* values for *t*-Student test. Legend: LUS—lung ultrasound; SD—standard deviation.

## Data Availability

The database of this study can be accessed upon request at the address adrian.toma@prof.utm.ro.
